# A Hospital Protocol for Decision Making in Emergency Admission for Acute Diverticulitis: Initial Results from Small Cohort Series

**DOI:** 10.3390/medicina56080371

**Published:** 2020-07-24

**Authors:** Paolo Ruscelli, Roberto Cirocchi, Alessandro Gemini, Paolo Bruzzone, Michelangelo Campanale, Massimiliano Rimini, Sergio Santella, Gabriele Anaia, Luigina Graziosi, Annibale Donini

**Affiliations:** 1 Department of Emergency Surgery, Azienda Ospedaliero Universitaria-Ospedali Riuniti Ancona, 60126 Ancona, Italy; ruscellipaolo@gmail.com (P.R.); mx.rimini@gmail.com (M.R.); sergio.santella@ospedaliriuniti.marche.it (S.S.); 2 Department of Surgery and Biomedical Sciences, University of Perugia, 06121 Perugia, Italy; roberto.cirocchi@unipg.it (R.C.); m.campanale93@gmail.com (M.C.); luiginagraziosi@yahoo.it (L.G.); annibale.donini@unipg.it (A.D.); 3 Department of General and Specialist Surgery “Paride Stefanini”, Sapienza University of Rome, 00161 Rome, Italy; paolo.bruzzone@uniroma1.it; 4 Department of Morphology, Experimental Medicine and Surgery, Section of Surgery 1, Sant’Anna Hospital, University of Ferrara, 44121 Ferrara, Italy; gabriele.anaia@unife.it

**Keywords:** acute diverticulitis, complicated diverticulitis, emergency surgery, non-operative treatment, percutaneous drainage, Hartmann’s procedure, colon resection

## Abstract

*Background and objectives*: We present initial results from a small cohort series for a hospital protocol related to the emergency hospitalization decision-making process for acute diverticulitis. We performed a retrospective analysis of 53 patients with acute diverticulitis admitted to the Department of Emergency and Trauma Surgery of the “Azienda Ospedaliero Universiaria-Ospedali Riuniti” in Ancona and to the Department of General and Emergency Surgery of the “Azienda Ospedaliera-Universitaria” in Perugia. *Materials and Methods:* All patients were evaluated according to hemodynamic status: stable or unstable. Secondly, it was distinguished whether patients were suffering from complicated or uncomplicated forms of diverticulitis. Finally, each patient was assigned to a risk class. In this way, we established a therapeutic/diagnostic process for each group of patients. *Results:* Non-operative treatment (NonOP) was performed in 16 patients, and it was successful in 69% of cases. This protocol primarily considers the patient’s clinical condition and the severity of the disease. It is based on a multidisciplinary approach, in order to implement the most suitable treatment for each patient. In stable patients with uncomplicated diverticulitis or complicated Hinchey grade 1 or 2 diverticulitis, the management is conservative. In all grade 3 and grade 4 forms, patients should undergo urgent surgery. *Conclusions:* This protocol, which is based on both anatomical damage and the severity of clinical conditions, aims to standardize the choice of the best diagnostic and therapeutic strategy for the patient in order to reduce mortality and morbidity related to this pathology.

## 1. Introduction

Diverticular disease is increasingly reported in Western industrialized countries and is primarily located in the left colon. Its incidence is approximately 30% in people aged 60 years or more and 60% in individuals aged 80 years or more [1]. Diverticulitis is the third most frequent cause of gastrointestinal disease requiring hospitalization [2,3], and it is the leading indication for elective colon resection [4]. Recently, an Italian national database study performed by Binda et al. reported a higher rate of hospitalization in patients less than 70 years old, especially those under 60; the hospitalization rate of patients ≥ 70 years was comparatively higher although unchanged [5,6]. There are three forms of diverticular disease: asymptomatic disease, symptomatic uncomplicated disease, and complicated disease [7].

Only 15% of patients with diverticulosis will develop, during their lifetime, acute diverticulitis, characterized by an inflammatory process affecting one or more diverticula [8]. Acute diverticulitis (AD) can range from simple forms with modest inflammation (uncomplicated) to complicated forms with pericolic or pelvic abscesses, perforations, and diffuse peritonitis [9,10].

These abscesses were reported in between 1% and 10% of all emergency hospital admissions for AD and 5–29% of cases with complicated AD; the rates of diffuse peritonitis ranged from 1.6% to 10.2% of all hospitalizations and from 11% to 47% of complicated cases [11].

In patients with intra-abdominal sepsis (IAS), the prognosis is poor [12,13,14] so the treatment of the diverticulitis is based on the severity of the disease. In the international literature, the Hinchey classification system is frequently used to stratify complicated acute diverticulitis [15,16].

The treatment of choice for acute diverticulitis in an emergency is still under debate [17]. Many guidelines have been published that differ both in the staging of the disease and in therapeutic indications [12,18,19,20,21,22,23].

Especially in cases of complicated diverticulitis, there is great variability in the diagnostic and therapeutic process, also considering surgical options [24,25,26,27,28,29,30,31,32,33]. In order to solve this problem, multidisciplinary protocols for diagnosis and treatment of diverticulitis have been proposed in common clinical practice, but these protocols were elaborated only for decision making for uncomplicated diverticulitis. For this reason, we proposed a protocol for global decision making for emergency hospital admission. This new protocol was based on the pathophysiological aspects that characterize acute diverticulitis and on the patient’s clinical condition. The primary objective of this study was to present the results after two years of implementing the protocol, considering the adhesion to it and the critical issues generated.

## 2. Materials and Methods

In 2017, a hospital protocol to treat acute colonic diverticulitis was approved by the institutional review board as a hospital-wide protocol and by the board of directors and the chief medical officer at “Azienda Ospedaliero Universitaria-Ospedali Riuniti” in Ancona. In the previous period, multidisciplinary team meetings of First Aid, Emergency Medicine, Radiology, Interventional Radiology, Gastroenterology, and Emergency Surgery developed the steps of this hospital protocol. 

Successively after the validation of the outcomes at “Azienda Ospedaliero Universitaria-Ospedali Riuniti” in Ancona, this protocol was adopted in common clinical practice at the Department of General and Emergency Surgery of “Azienda Ospedaliera-Universitaria” in Perugia. The protocol was archived in a repository of metadata records developed at the Emergency Surgical Department of “Azienda Ospedaliero Universitaria-Ospedali Riuniti” in Ancona. It is possible to write to the corresponding author to request a copy of this protocol in the Italian language. 

In this study, a retrospective analysis of the prospective database was performed. We included patients with acute complicated diverticulitis admitted consecutively at the Department of Emergency and Trauma Surgery of “Azienda Ospedaliero Universiaria-Ospedali Riuniti” in Ancona and at the Department of General and Emergency Surgery of “Azienda Ospedaliera-Universitaria” in Perugia, in the period between 1 January 2017 and 1 January 2019.

All subjects gave their informed consent for inclusion before they participated in the study. The study was conducted in accordance with the Declaration of Helsinki, and the protocol was approved by the Ethics Committee of Umbria (3142/17).

Variables analyzed were:

- Personal data (name, age, sex);
- Diagnosis at admission (all cases of AD);
- Degree of AD according to the World Society of Emergency Surgery (WSES) classification;
- Risk classes;
- Time of preoperative hospitalization;
- Time of postoperative hospitalization;
- Therapeutic strategy;
- Type of surgery;
- Outcome of non-operative treatment (NonOP);
- Outcome of the hospitalization.

In common hospital practice, patients with acute diverticular disease are evaluated according to hemodynamic status: stable or unstable. In frail and hemodynamically unstable patients, emergency surgical treatment should be as prompt as possible, regardless of Hinchey’s stage, and could include either Hartmann’s procedure, a Mikulicz stoma, or a damage control surgery (DCS) strategy [34]. In a hemodynamically stable patient, according to the type and severity of peritoneal contamination, there are two surgical options: either an immediate colonic resection, associated with primary anastomosis, protected or not from covering stoma, or Hartmann’s procedure.

In hemodynamically unstable patients with an acute abdomen due to suspicious complicated colon diverticulitis, treatment must be performed as soon as possible and therefore an abdominal computed tomography (CT) scan should be avoided; in these patients, diagnosis and evaluation of the severity of colon diverticulitis should be performed with an abdominal X-ray and “smart” abdominal ultrasonography (US) as the Focused Assessment with Sonography for Trauma (FAST). A careful radiological evaluation (US and/or CT scan) of AD is recommended in hemodynamically stable patients according to the World Society of Emergency Surgery (WSES) classification [19]. The uncomplicated forms are characterized by the presence of diverticula, thickening of the colon walls, or an increase in the density of pericolic fat.

The complicated ADs are classified into six degrees of WSES’ classification as reported in Table 1.

After a comprehensive evaluation, a risk class is assigned to each patient (Table 2).

Based on these evaluations, the subsequent therapeutic path is performed utilizing the WSES classification.

### 2.1. Acute Uncomplicated Diverticulitis

**Figure 1:**
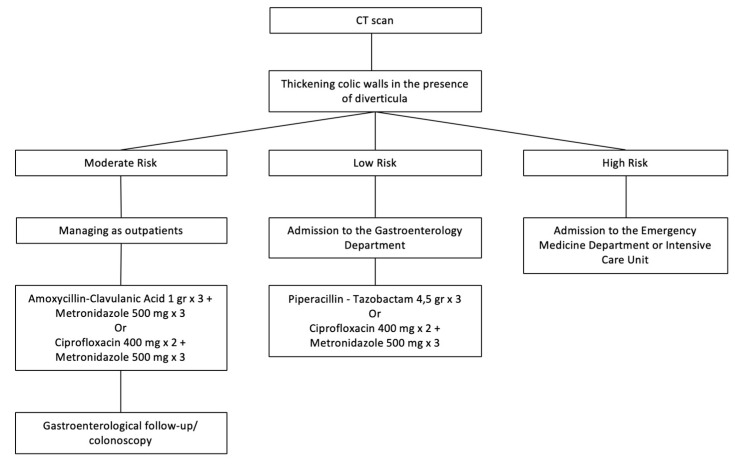
Management of acute uncomplicated diverticulitis. CT: computed tomography.

In the case of an uncomplicated acute diverticulitis, according to risk class, patients could be treated as outpatients with an antibiotic therapy (low and moderate risk classes) or admitted to an Emergency Medicine Department. After 4–6 weeks, a control colonoscopy is scheduled (Figure 1).

### 2.2. Acute Complicated Diverticulitis Grade 1A

**Figure 2:**
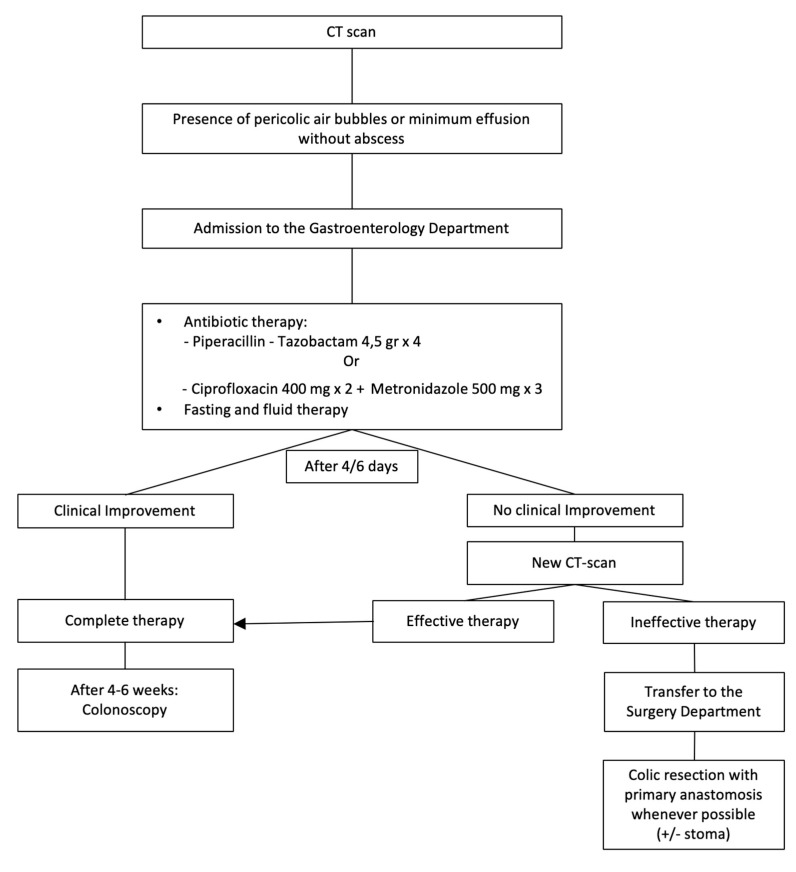
Management of acute complicated diverticulitis grade 1A.

Patients with grade 1A diverticulitis, characterized by the presence of pericolic air bubbles or a little pericolic fluid without abscess (within 5 cm of the inflamed bowel segment), are hospitalized in the Medical Department, possibly in a Gastroenterological Unit, to receive antibiotic therapy. In the absence of clinical and blood test improvement after 4–6 days, an abdominal US or CT scan is performed to evaluate the continuation of this therapeutic strategy or a possible surgical approach (colic resection with primary anastomosis as often as possible). After 4–6 weeks, a control colonoscopy is scheduled (Figure 2).

### 2.3. Acute Complicated Diverticulitis Grade 1B

**Figure 3:**
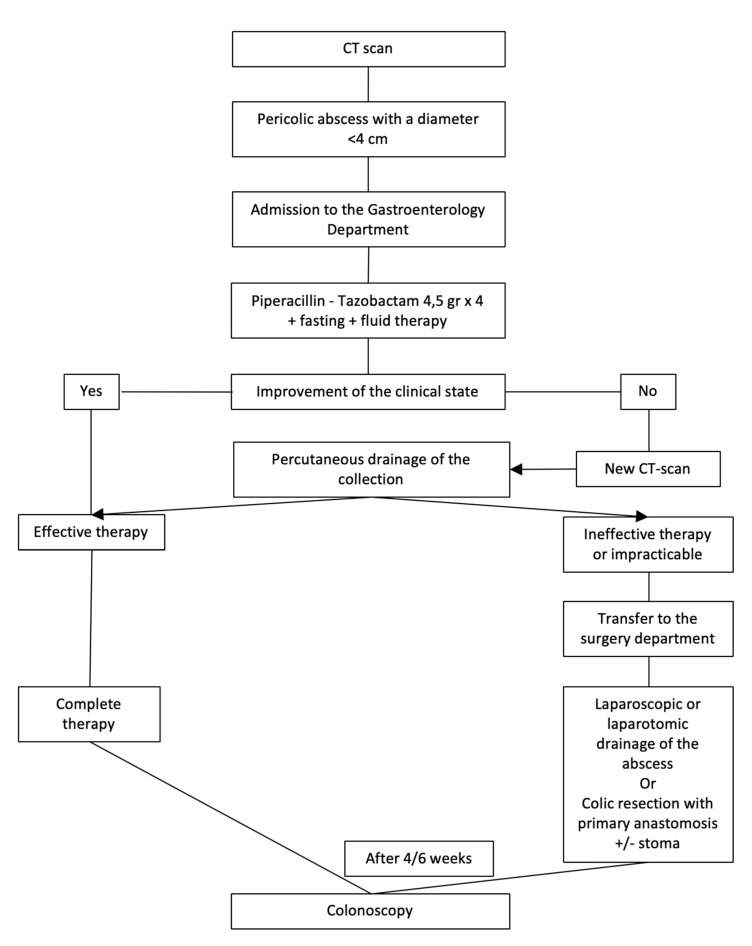
Management of acute complicated diverticulitis grade 1B.

Patients with grade 1B diverticulitis (presence of pericolic abscess with a diameter < 4 cm) are hospitalized in the Medical Department, if possible, in a Gastroenterological Unit, in order to receive intravenous therapy, obviously including antibiotics. In the absence of clinical and blood test improvement after 4–6 days, an abdominal CT scan is repeated. In the case of failure of medical therapy, the patient becomes a candidate for percutaneous drainage of the collection. If this is impossible or ineffective, the patient will receive surgery (laparoscopic or laparotomic drainage of the collection, or colic resection and primary anastomosis as often as possible). After 4–6 weeks, a control colonoscopy is scheduled (Figure 3).

### 2.4. Acute Complicated Diverticulitis Grade 2A

**Figure 4:**
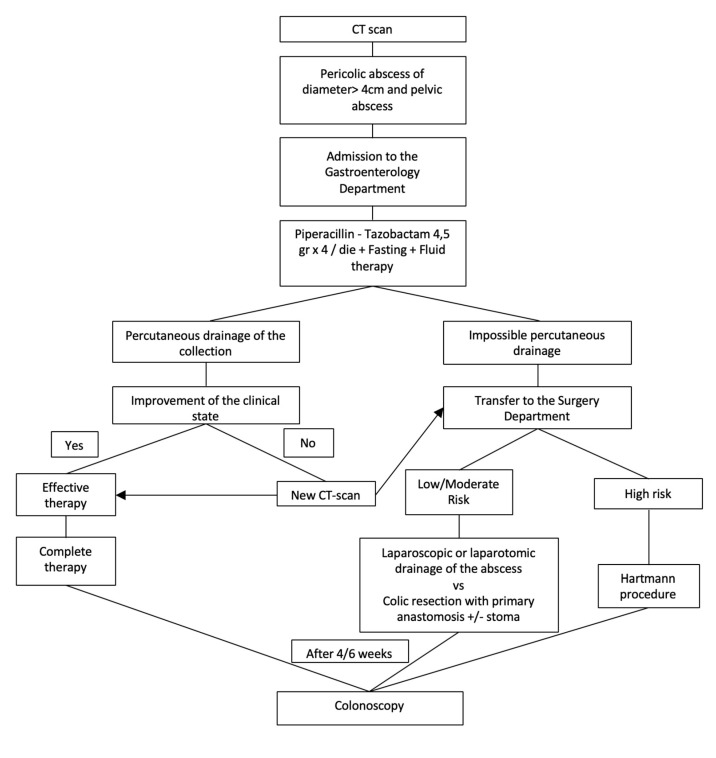
Management of acute complicated diverticulitis grade 2A.

Patients with grade 2A diverticulitis (pericolic abscess of diameter >4 cm and pelvic abscess) receive the same medical treatment with additional percutaneous drainage of the collection. If this is impossible, the patient should undergo open or laparoscopic surgery (drainage of collection or colonic resection with primary anastomosis as often as possible). Patients with a high-risk class, if candidates for surgery, should receive emergency intervention (Hartmann intervention). In the absence of clinical laboratory improvement after percutaneous drainage of the collection, an abdominal CT scan is repeated. According to the CT scan results, patients continue a medical-conservative approach, or undertake a surgical approach (laparoscopic or laparotomic drainage of the collection, or colic resection with primary anastomosis as often as possible). High-risk patients, if candidates for surgical treatment, should undergo emergency surgery (Hartmann’s procedure). After 4–6 weeks, a control colonoscopy is scheduled (Figure 4).

### 2.5. Acute Complicated Diverticulitis Grade 2B

**Figure 5:**
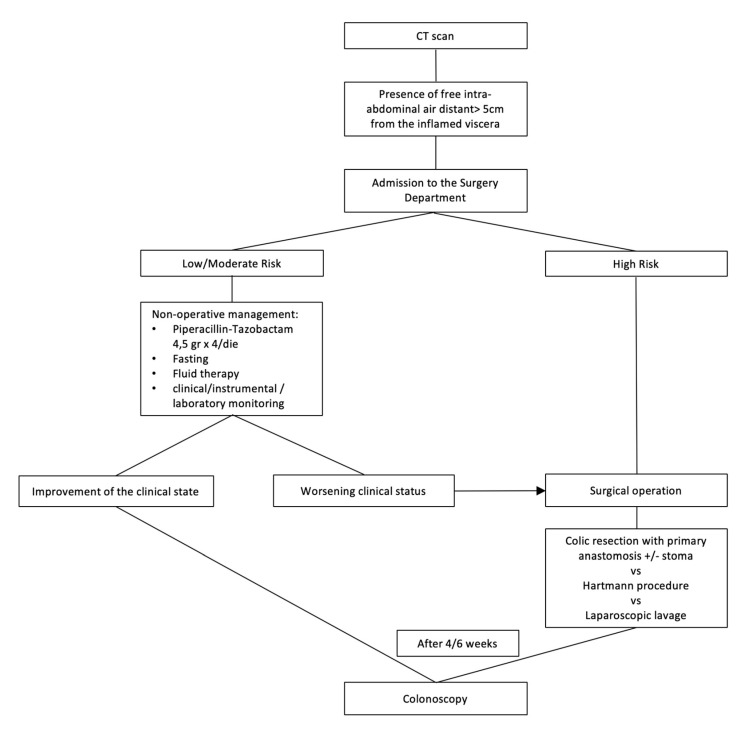
Management of acute complicated diverticulitis grade 2B.

The treatment of these patients (presence of free intra-abdominal air > 5 cm away from the inflamed viscera) is based on different risk classes of patients. Low or moderate risk class patients should receive a nonoperative treatment: close monitoring of clinical, laboratory, and instrumental parameters, and antibiotic therapy. In the case of worsening clinical status and in high risk class patients, treatment is the colic resection with primary anastomosis or Hartmann’s procedure; in very selected patients (low or moderate risk), a laparoscopic peritoneal lavage (LPL) and laparoscopic drainage may be indicated. After 4–6 weeks, a control colonoscopy is scheduled (Figure 5).

### 2.6. Acute Complicated Diverticulitis Grade 3

**Figure 6:**
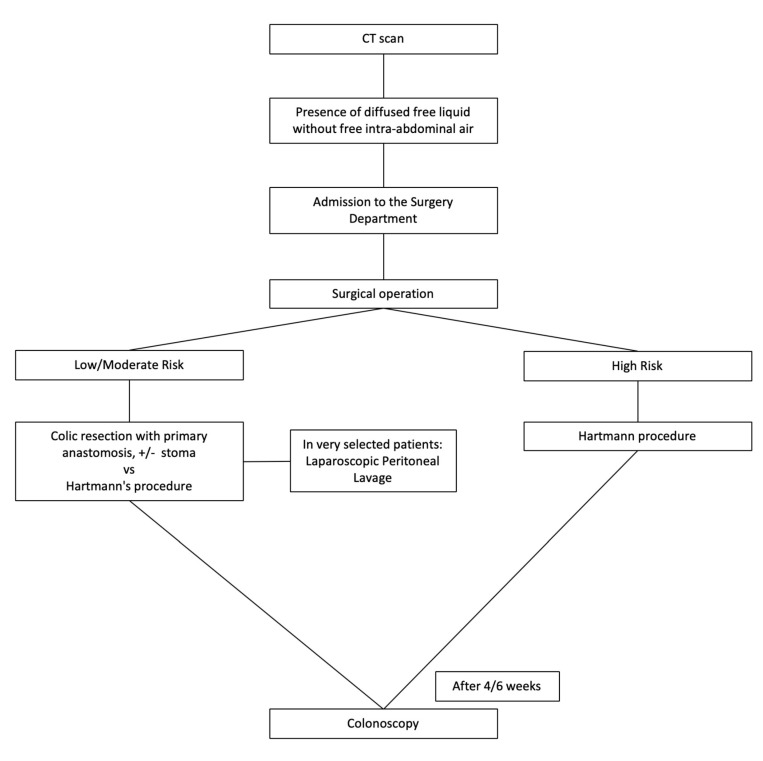
Management of acute complicated diverticulitis grade 3.

The treatment of these patients (presence of diffused free liquid without free intra-abdominal air) is based on different risk classes of patients. The most common treatment is urgent/emergency intervention (surgical resection and primary anastomosis or Hartmann’s procedure). In very selected patients (low or moderate risk), a laparoscopic peritoneal lavage (LPL) and laparoscopic drainage may be indicated; if it is not followed by clinical improvement, resective surgery is advised (Hartmann’s procedure or resection with primary anastomosis, protected or not by stoma). After 4–6 weeks, a control colonoscopy is scheduled (Figure 6).

### 2.7. Acute Complicated Grade 4 Diverticulitis Grade

**Figure 7:**
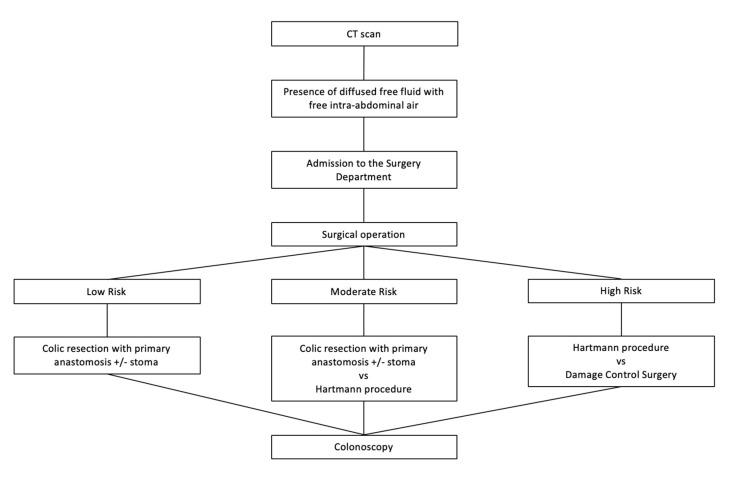
Management of acute complicated diverticulitis grade 4.

Patients with grade 4 diverticulitis (presence of diffused free liquid with free intra-abdominal air) should undergo surgery, and the type of surgical treatment is based on different risk classes of patients. Low risk class patients without significant comorbidities may undergo colic resection with primary anastomosis, protected or not from upstream stoma. Moderate risk class patients and those with a low risk class but with significant comorbidities should undergo Hartmann’s procedure. Patients with a high class of risk can be subject to DCS, followed by a second surgical look after 48–72 h in order to be able to pack the anastomosis and possibly close the abdominal wall fascia. These patients are admitted to the Intensive Care Unit (ICU) until the anastomosis is packaged, the laparotomy is closed, and the general conditions do not contraindicate the transfer to the surgical ward. After 4–6 weeks, a control colonoscopy is scheduled (Figure 7).

## 3. Results

**Figure 8:**
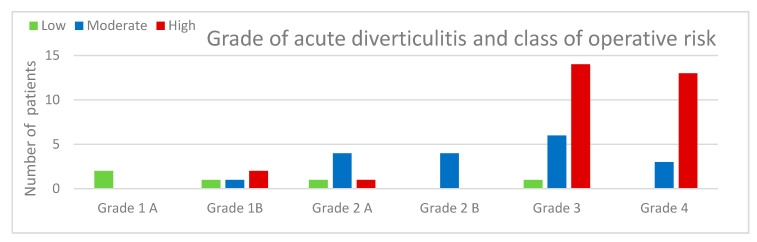
Number of patients on the basis of the grade of diverticulitis and the class of operative risk.

In the study period (1 January 2017–1 January 2019), 53 patients (pt) were studied. The mean age of our patients was 64 years (range 29–97 years) with a male/female (M/F) ratio of 1:1.5 (23 male and 30 female patients) (Table 3). On the basis of the new classification proposal from WSES, the patients who came to our observation were divided according to Table 4 (Table 4; Figure 8).

The results shown do not consider patients who were not hospitalized or those who spent one night in the hospital (“short observation” setting).

Regarding imaging exams, in hemodynamically stable patients (in total 48 patients), 23 patients underwent an abdominal ultrasound scan and CT scan, and 25 patients only CT scan; five patients with septic shock, hemodynamically unstable, underwent only abdominal X-ray and “smart” abdominal ultrasonography.

The high-risk patients frequently had acute complicated diverticulitis of grade 3 (24.5%) or 4 (24.5%).

Non-operative treatment (NonOP) was performed in 16 patients. It was performed in grade 1A (two cases), grade 1B (four cases), grade 2A (six cases), and grade 2B (four cases) patients. NonOP was successful in 69% of cases (11 patients). In half of the cases (eight patients: two patients 1A, two patients 1B, and four patients 2B), the treatment was only of a medical type (antibiotic therapy); meanwhile, in the other half of the cases, eight patients underwent percutaneous drainage (six patients in the 2A group and two patients in the 1B group) (Table 5).

NonOP at admission failed to control the clinical state in 5 of 16 patients: four patients in the group underwent percutaneous drainage (two patients in group 1B, both high risk; two patients in group 2A, one of whom was at high risk and one at moderate risk) and one patient in group 2B (at moderate risk) was treated with antibiotic therapy only.

Patients in whom NonOP failed underwent surgery: two patients in group 1B (one underwent laparoscopic peritoneal lavage (LPL) and the other underwent laparoscopic drainage of peritoneal abscess); two patients in group 2A after failure of percutaneous drainage underwent colic resection and anastomosis without ostomy; one patient in group 2B underwent Hartmann’s procedure.

In eight patients, abdominal percutaneous drainage was used to treat the abdominal abscess, as explained in the protocol: two patients in group 1B (those at high risk) and all patients in group 2A (six patients) (Table 5). The percutaneous drainage was successful in 50% of cases, while in the other half of the cases the patients then required surgery (two patients in group 1B and two patients in group 2A), as explained in the analysis of the patient in whom the NonOP treatment failed.

In thirty-seven patients (70%), emergency surgery was performed: 21 patients had complicated grade 3 acute diverticulitis, and the remaining 16 patients had grade 4 complicated AD. 

Examining patients with grade 3 acute complicated diverticulitis, these data emerged:

- A patient with low-risk grade 3 acute diverticulitis was treated with laparoscopic peritoneal lavage;
- Seven patients with grade 3 AD, in the moderate risk class, had the following different treatments: one patient received hemicolectomy with protective stomas, five patients underwent resection and anastomosis, and one patient underwent Hartmann’s procedure;
- Patients with high-risk acute grade 3 diverticulitis (13 cases) had the following treatments: three patients underwent Hartmann’s procedure; one underwent patient sub-total colectomy; and nine patients underwent resection and anastomosis.

In the end, examining all the 16 patients with grade 4 acute complicated diverticulitis, these data emerged:

- Five unstable patients underwent a smart evaluation that included abdomen X-ray and abdomen ultrasound only. All these patients were in high risk group. In these cases, the surgical procedures adopted were four Hartmann’s procedures and one DCS.
- Eleven stable patients underwent abdominal CT and then underwent Hartmann’s procedure (three moderate-risk patients and 8 high-risk patients).

## 4. Discussion

Incidence of AD and complicated AD has increased over the past 20 years [35,36,37]. Over the last few decades, there have been numerous changes in the choice of therapeutic approach for patients with acute diverticulitis. These changes have affected both medical and surgical therapy. Recent studies have shown the possibility of treating selected patients with medical therapy without the need for hospitalization; lately, You et al. published the results of NCT01986686. In this randomized controlled trial, a higher rate of enrolled patients following conservative management of diverticulitis with local extraluminal air did not require elective surgery (8% elective surgery versus 32% conservative management; *p* = 0.019) [38].

The surgical approach has changed over the years both in elective and emergency surgery, also thanks to the use of minimally invasive methods. Our protocol tries to implement the recent innovations introduced in the therapy of acute diverticulitis, starting from the adoption of standardized evaluation parameters.

In common clinical practice, it is important to distinguish acute diverticulitis into uncomplicated and complicated forms. This distinction is useful in order to establish which is the best hospital care setting and the best therapeutic treatment for a patient. Complicated diverticulitis can compromise the patient’s clinical condition and, in severe cases, can lead to septic shock. This category of patients should be quickly identified and subjected to intensive monitoring. On the other hand, patients in a stable clinical condition, suffering from uncomplicated diverticulitis, could benefit from home treatment [39]. In order to distinguish between complicated and uncomplicated forms of acute diverticulitis, in addition to the patient’s physical examination, laboratory tests, and an abdominal X-ray, the American Society of Colon and Rectal Surgeons (ASCRS) suggests that patients should undergo abdominal CT; CT scan in fact has a sensitivity of 98% and a specificity of 99%. Ultrasound (US) and magnetic resonance imaging (MRI) are considered an alternative in the evaluation of a patient with AD [40]. 

Recently, the ASCRS reported new guidelines in which it is recommended that: “CT scan of the abdomen and pelvis is the most appropriate initial imaging modality in the assessment of suspected diverticulitis. (Grade of Recommendation: Strong recommendation based on moderate-quality evidence, 1B)” [41]. The same recommendations were reported in the recent guidelines of WSES for management of acute left-sided colonic diverticulitis (ALCD): “In patients with suspected ALCD, we suggest contrast enhanced CT scan of the abdomen as imaging technique of first choice (weak recommendation based on moderate-quality evidence, 2B). We suggest to use US in the initial evaluation of patients with suspected ALCD if it is performed by an expert operator. It has wide availability and easy accessibility. A step-up approach with CT performed after an inconclusive or negative US may be a safe approach for patients suspected of acute diverticulitis (weak recommendation based on moderate-quality evidence, 2B)” [42].

Presently, there are numerous guidelines indicating the therapeutic approach for patients with acute diverticulitis. It should be noted that this disease, once considered mainly of surgical interest, is currently managed by surgeons, gastroenterologists, internists, and interventional radiologists [18,19,20,21,22,23].

Beyond the importance of imaging methods, we are convinced that the therapeutic decision must essentially be based on the evaluation of the patient’s clinical condition. In addition to hemodynamic stability, which immediately differentiates patients into two large groups, stable patients should be evaluated for: signs of peritonitis, CPR (C-reactive Protein) levels, increased heart rate, sensory changes, fever, and chills, in order to evaluate whether the patient must be hospitalized and in which department [43].

The decision-making process, due to the type of assessment and subsequent therapeutic choices as well as the references to the guidelines above, can also have significant repercussions in the malpractice field [44,45].

We have therefore implemented a treatment protocol that primarily considers the patient’s clinical condition and the severity of disease and that is based on a multidisciplinary approach in order to choose the most suitable treatment for the patient. First of all, we have divided patients into stable and unstable ones. Following the WSES indications, we divided the patients with acute diverticulitis into uncomplicated and complicated forms. The complicated forms were divided into six degrees of severity [22].

In our protocol, in stable patients with uncomplicated diverticulitis or complicated WSES grade 1 and 2 diverticulitis, the management is conservative. In patients with uncomplicated diverticulitis, the possibility of treating the patient at home may be considered [46,47]. In our experience, patients discharged at home are treated with oral antibiotic therapy and subsequent follow-up at a Gastroenterological Unit. However, some studies demonstrate the possibility of following patients at home without antibiotic therapy [48]. 

It is evident that acute diverticulitis is less and less a surgical disease. In fact, patients with colic abscess can also benefit from non-invasive treatment based on medical therapy and possible percutaneous drainage of the abscess. In our protocol, according to the literature, patients with an abscess smaller than 4 cm are subjected to medical therapy only. Patients who have a stable clinical condition and an abscess with a diameter greater than 4 cm, without the presence of air more than 5 cm away, are treated initially with percutaneous drainage and antibiotic therapy [22,33,41]. In the case of failure, a colonic resection is performed while if it is successful, surgery can be scheduled by reducing the colostomy rate.

In our protocol, patients suffering from high-risk grade 2A and 2B diverticulitis are subjected to surgery if the conservative strategy has not been effective or practicable. In all grade 3 and grade 4 forms, patients undergo urgent surgery. Therefore, in the group of patients treated surgically there are both patients who undergo emergency surgery and those who undergo scheduled surgery after the failure of the conservative strategy. The type of intervention is established on the basis of the patient’s clinical condition and on the basis of the intra-operative finding of the abdominal situation.

Depending on the general clinical condition, patients (in the low or medium risk class), even in the presence of perforation with free air in the abdomen, can be conservatively treated with medical therapy and possibly percutaneous drainage in order to subsequently plan an elective procedure, avoiding ostomy packaging. 

The mortality rate observed in our patients (5.8%), considering the small size of our sample, coincides with literature data (mortality rate between 6.3% and 10%, from 6% in purulent diverticulitis to 35% in stercoraceous perforation) [10,33,49,50].

The mean length of hospital stay in our patients is slightly higher than the literature data, with a mean stay of 11 days compared to 8 days in other studies [51].

Adherence to our new diagnostic and therapeutic protocol was 96%, increasing homogeneity of treatment among patients at the same stage and in comparable clinical conditions. According to our results, the objective is therefore also to encourage further studies to confirm the effectiveness of this new classification and of this new protocol, in order to also evaluate the rates of disease recurrence. One of the major limitations of our study is the small number of patients examined, due to the fact that our department admitted almost exclusively patients with acute high-grade acute diverticulitis or patients for whom the conservative treatment performed in medical departments was not successful. Finally, it should be noted that frail patients were also included in our protocol and were evaluated collegially (by anesthetists, surgeons, and internists) in order to establish the appropriateness of emergency surgical treatment.

## 5. Conclusions

A multidisciplinary diagnostic protocol has been defined and made operational in our clinical practice in order to guide physicians towards the best therapeutic diagnostic strategy for the patient and to homogenize the care choices aimed at reducing mortality and morbidity linked to this pathology. This protocol considers anatomic damage and severity of clinical condition.

It is important to follow a protocol to manage patients with acute diverticulitis by optimizing the indications and timing of surgical treatment and therefore increasing the chances of successful outcome. The high rate of adherence to the protocol shows that this is suitable even in an emergency setting.

More extensive and integrated studies, also in cooperation with gastroenterologists, could offer additional data on the basis of larger samples.

## Figures and Tables

**Table 1:** Classification of complicated acute diverticulitis sec. the World Society of Emergency Surgery (WSES) [19].

Grade 1A	presence of pericolic air bubbles or minimum effusion without abscess
Grade 1B	presence of pericolic abscess with a diameter < 4 cm
Grade 2A	pericolic abscess of diameter > 4 cm and pelvic abscess
Grade 2B	presence of free intra-abdominal air > 5 cm away from the inflamed viscera
Grade 3	presence of diffused free liquid without free intra-abdominal air
Grade 4	presence of diffused free fluid with free intra-abdominal air

**Table 2:** Risk classes of patients.

Low Risk	Patient Without a Higher Risk Class
Moderate risk	- White blood cells (WBC) count: > 10,000/mm^3^ or < 4000/mm^3^ - Body temperature (T) > 38 °C or < 36 °C
High risk	Sepsis complicated by the dysfunction of one or more of the following organs or systems: - Cardiovascular - Neurological - Respiratory - Liver - Hematological

**Table 3:** Characteristics of enrolled patients.

Male/female (n)	23/30
Age (years) mean	64
ASA score (mean)	3
Quick SOFA (mean)	1.7
CT scan (n. patients)	48
“Smart” ultrasonography evaluation (n. patients)	5
Non-operative treatment (n. patients)	16
Surgery as a first therapeutic approach (n. patients)	37
Intra-operative mortality	0%
Post-operative mortality	5.8%
Average total hospital stay (days)	11

ASA: American Society of Anesthesiologists; SOFA: Sequential Organ Failure Assessment; CT: Computed tomography.

**Table 4:** Patients divided according to the severity of disease, reported from WSES.

Acute complicated diverticulitis grade 1A	2 pt (3.7%)	Class of risk low	2 pt 3.7%
Acute complicated diverticulitis grade 1B	4 pt (7.5%)	Class of risk low	1 pt 1.9%
		Class of risk moderate	1 pt 1.9%
		Class of risk high	2 pt 3.7%
Acute complicated diverticulitis grade 2A	6 pt (11.3%)	Class of risk low	1 pt 1.9%
		Class of risk moderate	4 pt 7.5%
		Class of risk high	1 pt 1.9%
Acute complicated diverticulitis grade 2B	4 pt (7.5%)	Class of risk moderate	4 pt 7.5%
Acute complicated diverticulitis grade 3	21 pt (39.6%)	Class of risk low	1 pt 1.9%
		Class of risk moderate	7 pt 13.2%
		Class of risk high	13 pt 24.5%
Acute complicated diverticulitis grade 4	16 pt (30.2%)	Class of risk moderate	3 pt 5.7%
		Class of risk high	13 pt 24.5%

**Table 5:** Non-operative treatment.

NON-OPERATIVE TREATMENT (16 PATIENTS)			
ONLY ANTIBIOTIC THERAPY (8 PATIENTS)		PERCUTANEOUS DRAINAGE (8 PATIENTS)	
2 patients in group 1A	2 pt low risk	2 patients in group 1B	2 pt high risk
2 patients in group 1B	1 pt low risk	6 patients in group 2A	1 pt low risk
	1 pt moderate risk		4 pt moderate risk
4 patients in group 2B	4 pt moderate risk		1 pt high risk

## Abbreviations

Acute diverticulitis	(AD)
Number	(Nr.)
Damage control surgery	(DCS)
Ultrasonography	(US)
World Society of Emergency Surgery	(WSES)
Intensive Care Unit	(ICU)
American Society of Anesthesiologists	(ASA)
Patient	(pt)
Magnetic resonance imaging	(MRI)
Intra-abdominal sepsis	(IAS)
Non-operative treatment	(NonOP)
Computed tomography	(CT)
Focused Assessment with Sonography for Trauma	(FAST)
Laparoscopic peritoneal lavage	(LPL)
Male/female	(M/F)
Sequential Organ Failure Assessment	(SOFA)
American Society of Colon and Rectal Surgeons	(ASCRS)
Acute left-sided colonic diverticulitis	(ALCD)
C-reactive Protein	(CPR)

## References

[1] Dixon M.R., Trudel J.L., Cameron J.L. (2004). Diverticular disease of the colon. Current Surgical Therapy.

[2] Peery A.F., Dellon E.S., Lund J., Crockett S.D., McGowan C.E., Bulsiewicz W.J., Gangarosa L.M., Thiny M.T., Stizenberg K., Morgan U.R. (2012). Burden of gastrointestinal disease in the United States: 2012 update. Gastroenterology.

[3] Ellison D.L. (2018). Acute Diverticulitis Management. Crit. Care Nurs. Clin. N. Am..

[4] Shah S.D., Cifu A.S. (2017). Management of Acute Diverticulitis. JAMA.

[5] Binda G.A., Mataloni F., Bruzzone M., Carabotti M., Cirocchi R., Nascimbeni R., Gambassi G., Amato A., Vettoretto N., Pinnarelliet L. (2018). Trends in hospital admission for acute diverticulitis in Italy from 2008 to 2015. Tech. Coloproctol..

[6] Amato A., Mataloni F., Bruzzone M., Carabotti M., Cirocchi R., Nascimbeni R., Gambassi G., Vettoretto N.P., Pinnarelli L., Cuomo R. (2020). Hospital admission for complicated diverticulitis is increasing in Italy, especially in younger patients: A national database study. Tech. Coloproctol..

[7] Emile S.H., Elfeki H., Sakr A., Shalaby M. (2018). Management of acute uncomplicated diverticulitis without antibiotics: A systematic review, meta-analysis, and meta-regression of predictors of treatment failure. Tech. Coloproctol..

[8] Brook M.A., Victorino G.P., Harken A.H., Moore E. (2018). Diverticular disease of the colon. Abernathy’s Surgical Secrets.

[9] Krukowski Z.H., Matheson N.A. (1984). Emergency surgery for diverticular disease complicated by generalized and faecal peritonitis: A review. Br. J. Surg..

[10] Parisi A., Gemini A., Desiderio J., Petrina A., Trastulli S., Grassi V., Sani M., Pironi D., Santoro A. (2016). Laparoscopic peritoneal lavage: Our experience and review of the literature. Videosurgery Other Miniinvasive Tech..

[11] Cirocchi R., Popivanov G., Corsi A., Amato A., Nascimbeni R., Cuomo R., Annibale B., Konaktchieva M., Binda G.A. (2019). The Trends of Complicated Acute Colonic Diverticulitis—A Systematic Review of the National Administrative Databases. Medicina.

[12] Stollman N., Smalley W., Hirano I., Adams M.A., Dorn S.D., Dudley-Brown S.L., Flamm S.L., Gellad Z.F., Gruss C.B., Kosinski L.R. (2015). American Gastroenterological Association Institute Guideline on the Management of Acute Diverticulitis. Gastroenterology.

[13] Bielecki K., Kaminski P., Klukowski M. (2002). Large bowel perforation: Morbidity and mortality. Tech. Coloproctol..

[14] Ryan O.K., Ryan É.J., Creavin B., Boland M.R., Kelly M.E., Winter D.C. (2020). Systematic review and meta-analysis comparing primary resection and anastomosis versus Hartmann’s procedure for the management of acute perforated diverticulitis with generalised peritonitis. Tech. Coloproctol..

[15] Hinchey E.J., Schaal P.G., Richards G.K. (1978). Treatment of perforated diverticular disease of the colon. Adv. Surg..

[16] Kaiser A.M., Jiang J.-K., Lake J.P., Ault G., Artinyan A., Gonzalez-Ruiz C., Essani R., Beart R.W. (2005). The Management of Complicated Diverticulitis and the Role of Computed Tomography. Am. J. Gastroenterol..

[17] Shafi S., Priest E.L., Crandall M.L., Klekar C.S., Nazim A., Aboutanos M., Agarwal S., Bhattacharya B., Byrge N., Dhillon T.S. (2016). Multicenter validation of American Association for the Surgery of Trauma grading system for acute colonic diverticulitis and its use for emergency general surgery quality improvement program. J. Trauma Acute Care Surg..

[18] Jacobs D.O. (2007). Clinical practice. Diverticulitis. N. Engl. J. Med..

[19] Andersen J.C., Bundgaard L., Elbrønd H., Laurberg S., Walker L.R., Støvring J. (2012). Danish national guidelines for treatment of diverticular disease. Dan. Med. J..

[20] Vennix S., Morton D.G., Hahnloser D., Lange J.F., Bemelman W.A. (2014). The research committee of the European Society of Coloproctocology Systematic review of evidence and consensus on diverticulitis: An analysis of national and international guidelines. Colorectal Dis..

[21] Jackson J.D., Hammond T. (2014). Systematic review: Outpatient management of acute uncomplicated diverticulitis. Int. J. Colorectal Dis..

[22] Sartelli M., Catena F., Ansaloni L., Coccolini F., Griffiths E.A., Abu-Zidan F.M., Di Saverio S., Ulrych J., Kluger Y., Ben-Ishay O. (2016). WSES Guidelines for the management of acute left sided colonic diverticulitis in the emergency setting. World J. Emerg. Surg..

[23] Kruis W., Germer C.-T., Leifeld L. (2014). Diverticular Disease: Guidelines of the German Society for Gastroenterology, Digestive and Metabolic Diseases and the German Society for General and Visceral Surgery. Digestion.

[24] Fagenholz P.J., De Moya M.A. (2014). Acute Inflammatory Surgical Disease. Surg. Clin. N. Am..

[25] Wong D.W., Wexner S.D., Lowry A., Vernava A., Burnstein M., Denstman F., Fazio V., Kerner B., Moore R., Oliver G. (2000). Practice parameters for the treatment of sigmoid diverticulitis—Supporting documentation. Dis. Colon Rectum.

[26] Kohl A., Rosenberg J.B., Bock D., Bisgaard D., Skullman S., Thornell A., Gehrman J., Angenete E., Haglind E. (2018). Two-year results of the randomized clinical trial DILALA comparing laparoscopic lavage with resection as treatment for perforated diverticulitis. Br. J. Surg..

[27] Schwesinger W., Page C.P., Gaskill H.V., Steward R.M., Chopra S., Strodel W.E., Sirinek K.R. (2000). Operative management of diverticular emergencies: Strategies and outcomes. Arch. Surg..

[28] Brandt D., Gervaz P., Durmishi Y., Platon A., Morel P., Poletti P.A. (2006). Percutaneous CT Scan-Guided Drainage vs. Antibiotherapy Alone for Hinchey II Diverticulitis: A Case-Control Study. Dis. Colon Rectum.

[29] Sallinen V., Mentula P., Leppaniemi A.K. (2014). Nonoperative Management of Perforated Diverticulitis With Extraluminal Air Is Safe and Effective in Selected Patients. Dis. Colon Rectum.

[30] Liang S., Russek K., Franklin M.E. (2012). Damage control strategy for the management of perforated diverticulitis with generalized peritonitis: Laparoscopic lavage and drainage vs. laparoscopic Hartmann’s procedure. Surg. Endosc..

[31] Schultz J.K., Yaqub S., Wallon C., Blecic L., Forsmo H.M., Folkesson J., Buchwald P., Körner H., Dahl F.A., Øresland T. (2015). Laparoscopic Lavage vs. Primary Resection for Acute Perforated Diverticulitis. JAMA.

[32] Binda G.A., Karas J.R., Serventi A., Sökmen S., Amato A., Hydo L., Bergamaschi R., for the Study Group on Diverticulitis (2012). Primary anastomosisvsnonrestorative resection for perforated diverticulitis with peritonitis: A prematurely terminated randomized controlled trial. Colorectal Dis..

[33] Regenbogen S.E., Hardiman K.M., Hendren S., Morris A.M. (2014). Surgery for Diverticulitis in the 21st Century. JAMA Surg..

[34] Cirocchi R., Nascimbeni R., Binda G.A., Vettoretto N., Cuomo R., Gambassi G., Amato A., Annibale B. (2020). Surgical treatment of acute complicated diverticulitis in the elderly. Minerva Chir..

[35] Wheat C.L., Strate L.L. (2015). Trends in Hospitalization for Diverticulitis and Diverticular Bleeding in the United States From 2000 to 2010. Clin. Gastroenterol. Hepatol..

[36] Sartelli M., Moore F.A., Ansaloni L., Di Saverio S., Coccolini F., Griffiths E.A., Coimbra R., Agresta F., Sakakushev B., Ordóñez C. (2015). A proposal for a CT driven classification of left colon acute diverticulitis. World J. Emerg. Surg..

[37] Weizman A.V., Nguyen G.C. (2011). Diverticular disease: Epidemiology and management. Can. J. Gastroenterol. Hepatol..

[38] You J.K., Bendl R., Taut C., Sullivan R., Gachabayov M., Bergamaschi R., the Study Group on Diverticulitis (2018). Randomized clinical trial of elective resection versus observation in diverticulitis with extraluminal air or abscess initially managed conservatively. Br. J. Surg..

[39] Cirocchi R., Randolph J.J., Binda G.A., Gioia S., Henry B.M., Tomaszewski K.A., Allegritti M., Arezzo A., Marzaioli R., Ruscelli P. (2019). Is the outpatient management of acute diverticulitis safe and effective? A systematic review and meta-analysis. Tech. Coloproctol..

[40] Feingold D., Steele S., Lee S., Kaiser A., Boushey R., Buie W.D., Rafferty J.F. (2014). Practice Parameters for the Treatment of Sigmoid Diverticulitis. Dis. Colon Rectum.

[41] Hall J., Hardiman K., Lee S., Lightner A., Stocchi L., Paquette I.M., Steele S.R., Feingold D.L., Prepared on Behalf of the Clinical Practice Guidelines Committee of the American Society of Colon and Rectal Surgeons (2020). The American Society of Colon and Rectal Surgeons Clinical Practice Guidelines for the Treatment of Left-Sided Colonic Diverticulitis. Dis. Colon Rectum.

[42] Sartelli M., Weber D.G., Kluger Y., Ansaloni L., Coccolini F., Abu-Zidan F., Augustin G., Ben-Ishay O., Biffl W.L., Bouliaris K. (2020). 2020 update of the WSES guidelines for the management of acute colonic diverticulitis in the emergency setting. World J. Emerg. Surg..

[43] Biondo S. (2019). The diminishing role of surgery for acute diverticulitis. Br. J. Surg..

[44] Popivanov G., Fedeli P., Cirocchi R., Lancia M., Mascagni D., Giustozzi M., Teodosiev I., Kjossev K., Konaktchieva M. (2020). Perirectal Hematoma and Intra-Abdominal Bleeding after Stapled Hemorrhoidopexy and STARR—A Proposal for a Decision-Making Algorithm. Medicina.

[45] Chiarini S., Ruscelli P., Cirocchi R., D’Andrea V., Sensi B., Santoro A., Corsi A., Zepponi F., Fedeli P., Gioia S. (2020). Intersigmoid Hernia: A Forgotten Diagnosis—A Systematic Review of the Literature over Anatomical, Diagnostic, Surgical, and Medicolegal Aspects. Emerg. Med. Int..

[46] Horesh N., Wasserberg N., Zbar A.P., Gravetz A., Berger Y., Gutman M., Rosin D., Zmora O. (2016). Changing paradigms in the management of diverticulitis. Int. J. Surg..

[47] Swanson S.M., Strate L.L. (2018). Acute Colonic Diverticulitis. Ann. Intern. Med..

[48] Balasubramanian I., Fleming C., Mohan H.M., Schmidt K., Haglind E., Winter D.C. (2016). Out-Patient Management of Mild or Uncomplicated Diverticulitis: A Systematic Review. Dig. Surg..

[49] Stollman N., Raskin J.B. (2004). Diverticular disease of the colon. Lancet.

[50] Schmidt S., Ismail T., Puhan M.A., Soll C., Fuchs J. (2018). Meta-Analysis of surgical strategies in perforated left colonic diverticulitis with generalized peritonitis. Langenbeck’s Arch. Surg..

[51] Reynolds I.S., O’Connell E., Heaney R.M., Khan W., Waldron R., Barry K. (2017). Adherence to clinical guidelines and the potential economic benefits of admission avoidance for acute uncomplicated diverticulitis. Ir. J. Med. Sci..

